# Professional fulfillment in interventional radiology

**DOI:** 10.1186/s42155-025-00588-1

**Published:** 2025-11-08

**Authors:** Lindsay Eysenbach, Mark Loper, Gabe Li, David S. Shin, Eric J. Monroe, Matthew Abad-Santos, Eunjee Lee, Hyeonjeong Lim, Anthony Hage, Jeffrey Forris Beecham Chick, Mina S. Makary

**Affiliations:** 1 Section of Vascular and Interventional Radiology, Department of Radiology, University of Washington, 1107 NE 45th St, Seattle, WA 98105 USA; 2 Division of Vascular and Interventional Radiology, Department of Radiology, The Ohio State University Wexner Medical Center, 410 W 10th Ave, Columbus, OH 43210 USA; 3 Division of Vascular and Interventional Radiology, Department of Radiology, University of Southern California, 1500 San Pablo St, Los Angeles, California 90033 USA; 4 Division of Interventional Radiology, Department of Radiology, University of Wisconsin, 600 Highland Ave, Madison, WI 53792 USA; 5 Department of Information and Statistics, Chungnam National University, 99 Daehak-ro, Yuseong-Gu, Daejeon 34134 South Korea; 6 Section of Vascular and Interventional Radiology, Department of Radiology, 1 Cooper Plaza, Camden, NJ 08103 USA

**Keywords:** Professional fulfillment, Satisfaction, Burnout, Interventional radiology

## Abstract

**Background:**

There have been several analyses conducted demonstrating a sharp decrease in general physician fulfillment and satisfaction. Other studies have demonstrated that burnout, anxiety, and moral injury are prevalent among interventional radiologists specifically, however there is a paucity of literature examining professional fulfillment within the profession. The purpose of this study was to characterize professional fulfillment through job, career, and specialty satisfaction scores among interventional radiologists using a validated assessment tool.

**Results:**

There were 106 respondents included in the analysis: 97 (91.5%) practicing interventional radiologists and 9 (8.5%) interventional radiology trainees, including 87 (82.1%) males and 19 (17.9%) females. Respondents included those in academic (40; 37.7%), private practice (46; 43.4%), and hybrid/other settings (20; 18.9%), as well as at various lengths of practice. The mean job satisfaction score was 3.48, with 38 (35.8%) of respondents expressing a mean score of ≥ 4, which has been established as being “satisfied”. The mean career satisfaction score was 3.40, with 38 (35.8%) of respondents reporting a mean score of ≥ 4. The mean global specialty satisfaction was 3.63 with 53 (50.0%) of respondents reporting a mean score of ≥ 4.

**Conclusions:**

Professional fulfillment is low among interventional radiologists, with half expressing global specialty satisfaction and with minority percentages signaling job and career satisfaction. Patient interaction and work-life balance were identified as significant factors positively affecting professional fulfillment.

## Background

Professional fulfillment is made up of the levels of satisfaction one holds in their job, career, and specialty. As described by Brown & Gunderman, satisfaction is a term used for an activity being sufficient on a surface level, whereas fulfillment is a holistic feeling of accomplishment and completion [1]. Among physicians, these intersecting ideas have garnered attention due to their direct association with healthcare outcomes, patient happiness, and physician burnout. As an example, a survey conducted by Al Rekabi et al., similar to the one detailed here, found an increase in burnout among UK-based interventional radiologists alongside high levels of emotional exhaustion, depersonalization, and lack of personal accomplishment [2]. Follow-up dialogues regarding this important paper lend astute insight into potential alternate approaches and solutions of this complex problem, such as the differing yet synergistic views of Uberoi et al. and Reekers on the importance of increasing workforce in the field vs recognizing the lack of independence of the field itself and how that contributes a multidomain approach to burnout [3, 4]. Other previous works in addition to these conclude a physician’s satisfaction and well-being, or lack thereof, permeates into his or her encounters with patients, thus affecting more than solely the provider’s mood [1, 5–8]. Professional fulfillment considers additional factors alongside burnout in addressing this growing problem. Various frameworks have been validated to characterize and assess professional fulfillment as it pertains to physicians specifically separate from prior burnout assessments [5]. Specialty-agnostic assessment tools that define specific domains of physician experiences (relationships with patients, pay, administration, etc.) have been utilized to examine how these individual domains correlate with overall job, career, and specialty satisfaction [9, 10]. 

There have been several analyses conducted demonstrating a sharp decrease in general physician fulfillment and satisfaction [5]. Other studies have demonstrated that burnout, anxiety, and moral injury are prevalent among interventional radiologists specifically, however there is a paucity of literature examining professional fulfillment within the profession [11–13]. The aim of this study was to characterize professional fulfillment, as well as its potential origins, among interventional radiologists using a validated assessment tool.

## Methods

### Study population and data collection

This *Health Insurance Portability and Accountability Act (HIPAA)*-compliant study was exempt from *Institutional Review Board (IRB)* approval based on institutional assessment of criteria listed in 45 CFR 46.101(b). The study was assessed using STrengthening the Reporting of OBservational studies in Epidemiology (STROBE) guidelines [14]. This survey was disseminated between September 23, 2023 and October 14, 2023 (22 days). The survey was shared multiple times using social media platforms (i.e. X, San Francisco, CA; Facebook, Menlo Park, CA; LinkedIn, Sunnyvale, CA) and society-wide communications (Society of Interventional Radiology Connect, Fairfax, VA). Multiple direct contacts via electronic mail augmented recruitment efforts.

### Survey design and evaluation

An anonymous 54-question survey was created using Google Forms (Google; Mountain View, CA). The first section contained 11 questions related to demographics and practice environments. The second section contained 42 questions using a 5-point Likert scale from a previously validated physician satisfaction survey [9, 10]. Following the model of previous researchers [15], six questions not applicable to interventional radiology were removed for clarity (e.g. “Formularies or prescription limits restrict the quality of care I provide”). The final component of the survey included eight specific domains which contribute to patient satisfaction (i.e. relationships with colleagues, relationships with non-physician staff, relationships with patients, personal time, pay, autonomy, administration, and resources), as well as global measures of job, career, and specialty satisfaction. There was also an optional opportunity for open-ended comments at the end of the survey. Practicing interventional radiologists and current interventional radiology trainees were eligible to participate in the study. There were no incomplete submissions as answers to all questions except an optional free-response question were mandatory for survey submission.

### Measures and definitions

For the purposes of this study, and in keeping with prior literature, the term professional fulfillment refers to an umbrella term consisting of three individual measures: job satisfaction, career satisfaction, and specialty satisfaction [16]. Job satisfaction refers to the extent to which an individual is satisfied with their current job [16]. Career satisfaction refers to satisfaction with general career trajectory (i.e. work-related experiences). Specialty satisfaction refers to satisfaction with medical specialty (i.e. interventional radiology) [16].

### Statistical analyses

Statistical analyses were performed by two statisticians (EL, HL). Cronbach's alpha was calculated for questions within each satisfaction domain, with Cronbach’s alpha > 0.70 considered adequate reliability. There were three domains with Cronbach’s alpha < 0.70 (autonomy, administration, and resources); therefore, each question in this domain was analyzed separately (Table 1).

**Table 1:** Cronbach's alpha

Category	Cronbach’s alpha
Autonomy	0.58
Personal time	0.77
Relationships with patients	0.77
Relationships with colleagues	0.84
Relationships with staff	0.86
Pay	0.74
Administration	0.59
Resources	0.59
Global job satisfaction	0.86
Global career satisfaction	0.89
Global specialty satisfaction	0.91

Mean satisfaction score was analyzed for each professional fulfillment domain, and a satisfaction “ratio” was generated. A satisfaction score of 4 or 5 on a 5-point Likert scale was considered “satisfied,” based on prior work [17].

Pearson’s correlation analysis was performed between each satisfaction domain and global measures of satisfaction, with *p* < 0.05 considered statistically significant. Univariate logistic regression was fitted to see the relationship between job satisfaction and relevant variables. To resolve a false positive problem in the multiple testing, Bonferroni correction was performed, and the significance level was adjusted as 0.00385.

Finally, multivariate logistic regression was performed. Some variables were excluded due to high correlation and explored fitting for a full model. However, interpreting regression coefficients became challenging, particularly due to values such as “prefer not to say” in the gender variable, which essentially represent missing data. Therefore, analysis was conducted for the remaining 112 individuals, excluding those who chose “prefer not to say”. Additionally, “unsure” and “no” responses for the “Do you have an institutional practice…” item were combined to create a broader yes/no category. After identifying that the coefficient and odds ratio values for “International” in the “Location of practice” variable were exceptionally large, six observations were excluded to facilitate interpretation. After these refinements, the final number of respondents included in the analysis was 106.

## Results

### Demographics

Of 106 respondents included in the analysis, 97 (91.5%) were practicing interventional radiologists and 9 (8.5%) were interventional radiology trainees. Ninety-three (82.1%) of the respondents were male, and the majority (*n* = 63, 59.4%) were white. Respondents included practicing interventional radiologists from different career stages: 21 (19.8%) with practice experience of < 5 years, 19 (17.9%) with 6–10 years, 20 (18.9%) with 11–20 years, and 36 (34.0%) with > 20 years. *Additional demographic data are shown in *Table 2*.*

**Table 2:** Demographic and practice characteristics

	**Category**	**N (%)**
Current level of practice	Radiology trainee	9 (8.5%)
	Radiologist	97 (91.5%)
Gender	Female	19 (17.9%)
	Male	93 (82.1%)
Ethnicity	Asian	21 (19.8%)
	White	63 (59.4%)
	Others	23 (20.8%)
Location of practice	Midwest	23 (21.7%)
	Northeast	25 (23.6%)
	Southeast	20 (18.0%)
	Southwest	11 (10.4%)
	West	27 (25.5%)
Practice setting	Academic	40 (37.7%)
	Hybrid/Other	20 (18.9%)
	Private	46 (43.4%)
Practice in > 1 hospital/setting?	Yes	69 (65.1%)
	No	37 (34.9%)
Institutional leadership position?	Yes	59 (55.7%)
	No	47 (44.3%)
Years in practice	Trainee	10 (9.4%)
	< 5 years	21 (19.8%)
	6–10 years	19 (17.9%)
	11–20 years	20 (18.9%)
	> 20 years	36 (34.0%)
Time spent reading diagnostic studies	< 25%	37 (34.9%)
	25–49%	19 (17.9%)
	50–75%	3 (2.8%)
	> 75%	1 (0.9%)
	None	46 (43.4%)
Time dedicated to administrative tasks	< 25%	66 (62.3%)
	25–49%	11 (10.4%)
	None	29 (27.4%)

### Practice environments

Respondents practiced in academic (*n* = 40, 37.7%), private (*n* = 46, 43.4%), and hybrid/other (*n* = 20, 18.9%) environments. A majority of respondents (*n* = 60, 56.6%) did spend some time reading diagnostic studies, but only 4 (3.8%) spent > 50% of their time reading diagnostic studies. A majority (*n* = 59, 55.7%) reported holding leadership positions. A larger majority (*n* = 77, 72.6%) spent at least some time on administrative duties, with 13 (12.6%) reported spending > 25% of their time on these duties. *Additional practice characteristics data are shown in *Table 2*.*

### Professional fulfillment

**Fig. 1:**
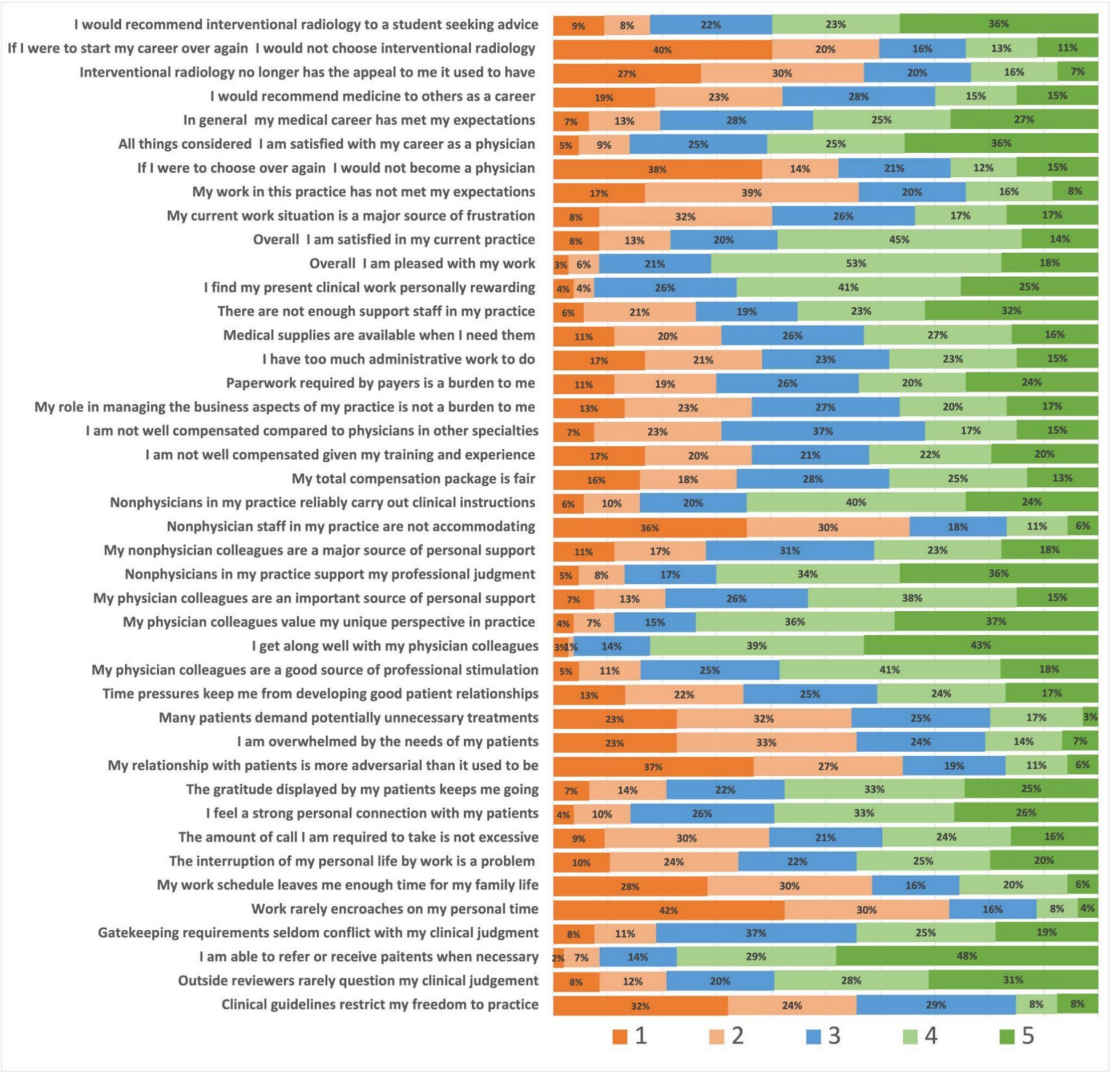
Likert scale results by query in second section of survey

Mean global job satisfaction score was 3.48; 38 (35.8%) of respondents reported a satisfaction score of ≥ 4. Mean global career satisfaction score was 3.40; 38 (35.8%) of respondents reported a satisfaction score ≥ 4. The mean global specialty satisfaction score was 3.62, with 53 (50.0%) respondents reporting satisfaction score ≥ 4. *Full non-demographic, non-practice-related survey results can be seen in *Fig. 1*. *

### Univariate analysis

Correlation analysis demonstrated a positive correlation between job, career, and specialty satisfaction and the composite satisfaction domains with high reliability (i.e. Cronbach’s alpha > 0.70): relationships with colleagues, relationships with staff, relationships with non-physician staff, personal time, and pay. *Additional results are shown in *Table 3.

**Table 3:** Pearson’s correlation analysis

	Global job satisfaction	Global career satisfaction	Global specialty satisfaction
Relationships with colleagues	0.60(< 0.001)*	0.46(< 0.001)*	0.48(< 0.001)*
Relationships with patients	0.60(< 0.001)*	0.63(< 0.001)*	0.53(< 0.001)*
Relationships with non-physician staff	0.55(< 0.001)*	0.36(< 0.001)*	0.44(< 0.001)*
Personal time	0.54(< 0.001)*	0.48(< 0.001)*	0.31(< 0.001)*
Pay	0.48(< 0.001)*	0.46(< 0.001)*	0.28(< 0.001)*
Autonomy			
Clinical guidelines restrict my freedom to practice	−0.21(0.03)	−0.24(0.01)	−0.05(0.64)
Outside reviewers rarely question my professional judgements	0.32(< 0.001)*	0.34(< 0.001)*	0.21(0.03)
I am able to refer patients or receive referrals when necessary	0.30(< 0.001)*	0.31(< 0.001)*	0.29(< 0.001)*
Gatekeeping requirements seldom conflict with my clinical judgment	0.22(0.03)	0.17(0.08)	0.08(0.42)
Administration			
My role in managing the business aspects of my practice is not a burden to me	0.34(< 0.001)*	0.31(< 0.001)*	0.32(< 0.001)*
Paperwork required by payers is a burden to me	−0.28(0.01)	−0.35(< 0.001)*	−0.28(< 0.001)*
I have too much administrative work to do	−0.38(< 0.001)*	−0.43(< 0.001)*	−0.38(< 0.001)*
Resources			
Medical supplies are available when I need them	0.44(< 0.001)*	0.23(0.03)	0.27(< 0.001)*
There are not enough support staff in my practice	−0.28(< 0.001)*	−0.17(0.05)	−0.07(0.50)

^*^Significant result. To resolve a false positive problem in the multiple testing, Bonferroni correction was performed and the significance level was adjusted to *p* < 0.0036

Logistic regression demonstrated a positive correlation between job, career, and specialty satisfaction and all composite domains analyzed, with the exception of career satisfaction and relationship with non-physician staff. *Additional results are shown in *Table 4*.*

**Table 4:** Logistic regression (Odds ratio)

	**Global job satisfaction**	**Global career satisfaction**	**Global specialty satisfaction**
Relationships with colleagues	1.42(< 0.001)*	1.38(< 0.001)*	1.35(< 0.001)*
Relationships with patients	1.33(< 0.001)*	1.40(< 0.001)*	1.31(< 0.001)*
Relationships with non-physician staff	1.26(< 0.001)*	1.15(0.016)	1.21(< 0.001)*
Personal time	1.35(< 0.001)*	1.28(< 0.001)*	1.20(0.002)*
Pay	1.40(< 0.001)*	1.35(< 0.001)*	1.20(0.008)*
Clinical guidelines restrict my freedom to practice	0.60(0.009)	0.76(0.126)	1.07(0.689)
Outside reviewers rarely question my professional judgements	2.01(< 0.001)*	1.33(0.092)	1.25(0.150)
I am able to refer patients or receive referrals when necessary	1.25(0.298)	1.67(0.030)	1.35(0.131)
Gatekeeping requirements seldom conflict with my clinical judgment	1.56(0.023)	1.40(0.078)	1.11(0.550)
My role in managing the business aspects of my practice is not a burden to me	0.42(0.011)*	0.42(0.011)*	0.46(0.004)*
Paperwork required by payers is a burden to me	0.74(0.053)	0.65(0.008)	0.80(0.142)
I have too much administrative work to do	0.69(0.021)	0.50(< 0.001)*	0.62(0.003)*
Medical supplies are available when I need them	2.18(< 0.001)*	1.28(0.148)	1.41(0.037)
There are not enough support staff in my practice	0.71(0.032)	0.78(0.125)	0.89(0.449)

^*^Significant result. To resolve a false positive problem in the multiple testing, Bonferroni correction was performed and the significance level was adjusted to *p* < 0.0036

### Multivariate logistic regression

In terms of demographics, no variables met statistical significance on multivariate analysis. There was a positive correlation between global job satisfaction and domains of personal time (*p* = 0.017) and relationships with patients (*p* = 0.034); there was also a statistically significant relationship with two questions within the “autonomy domain” (“Clinical guidelines restrict my freedom to practice,” “Outside reviewers rarely question my professional judgements”). There was a positive trend between global job satisfaction and pay that did not meet statistical significance (*p* = 0.064).

Similarly, there was a positive correlation between global career satisfaction and personal time (*p* = 0.043) and relationship with patients (*p* = 0.034); there was also a statistically significant correlation with pay (*p* = 0.047). Analysis of global specialty satisfaction only found a statistically significant correlation with relationships with patients (*p* = 0.001). *Full results are available in *Tables 5, 6 and 7*.*

**Table 5:** Multivariate logistic regression: global job satisfaction

	**Coefficient**	**Odds-ratio**	***p*-value**
Clinical guidelines restrict my freedom to practice	−1.933	0.14	0.042*
Gatekeeping requirements seldom conflict with my clinical judgment	−0.967	0.38	0.395
I am able to refer patients or receive referrals when necessary	−0.436	0.65	0.418
Outside reviewers rarely question my professional judgements	1.915	6.79	0.037*
Personal time	3.379	29.3	0.017*
Relationships with patients	3.174	23.9	0.034*
Relationships with colleagues	0.015	1.01	0.988
Relationships with staff	0.581	1.79	0.673
Pay	2.165	8.72	0.064
My role in managing the business aspects of my practice is not a burden to me	0.522	1.69	0.315
Paperwork required by papers is a burden to me	0.482	1.62	0.460
I have too much administrative work to do	0.657	1.93	0.322
Medical supplies are available when I need them	0.107	1.11	0.867
There are not enough support staff in my practice	−0.369	0.69	0.543
Current level of practice (practicing interventional radiologist)	−5.625	0.00	0.101
Gender (Male)	2.665	14.4	0.157
Ethnicity (Others)	−0.086	0.92	0.968
Ethnicity (White)	−0.740	0.48	0.735
Location of Practice			
*Northeast*	−0.473	0.62	0.800
*Southeast*	−2.844	0.06	0.291
*Southwest*	0.498	1.64	0.822
*West*	−1.177	0.31	0.586
Description of practice			
*Hybrid* + *Other*	−2.933	0.05	0.235
*Private*	−0.871	0.42	0.640
Practicing in more than one hospital setting (Yes)	2.593	13.37	0.174
Having an institutional practice leadership position (Yes)	3.984	53.74	0.048
How many years have you been in practice	0.008	1.01	0.943
What percentage of your daily work is reading diagnostic studies			
> *75%*	−1.381	0.25	1.000
*25–49%*	1.308	2.82	0.537
*50–75%*	3.597	36.50	0.902
*None*	4.426	83.59	0.085
What percentage of your time is dedicated to administrative non clinical duties			
*25–49%*	−5.367	0.00	0.110
*None*	−1.945	0.14	0.343

**Table 6:** Multivariate logistic regression: global career satisfaction

	**Coefficient**	**Odds-ratio**	***p*-value**
Clinical guidelines restrict my freedom to practice	0.549	1.73	0.276
Gatekeeping requirements seldom conflict with my clinical judgment	−0.324	0.72	0.671
I am able to refer patients or receive referrals when necessary	0.524	1.69	0.299
Outside reviewers rarely question my professional judgements	−0.858	0.42	0.070
Personal time	1.502	4.49	0.043*
Relationships with patients	3.174	23.9	0.034*
Relationships with colleagues	1.731	5.64	0.148
Relationships with staff	−0.383	0.68	0.596
Pay	1.303	3.68	0.047*
My role in managing the business aspects of my practice is not a burden to me	0.719	2.05	0.114
Paperwork required by papers is a burden to me	−0.627	0.53	0.200
I have too much administrative work to do	−0.493	0.67	0.382
Medical supplies are available when I need them	−0.619	0.54	0.219
There are not enough support staff in my practice	−0.218	0.80	0.672
Current level of practice (practicing interventional radiologist)	−0.114	0.32	0.639
Gender (Male)	0.480	1.62	0.686
Ethnicity (Others)	−0.316	0.04	0.088
Ethnicity (White)	−0.453	0.01	0.023
Location of Practice			
*Northeast*	1.189	3.28	0.023
*Southeast*	1.189	3.28	0.427
*Southwest*	1.675	5.34	0.252
*West*	0.885	2.42	0.626
Description of practice			
*Hybrid* + *Other*	0.793	2.21	0.653
*Private*	−0.251	0.08	0.155
Practicing in more than one hospital setting (Yes)	−0.334	0.71	0.751
Having an institutional practice leadership position (Yes)	1.952	7.04	0.071
How many years have you been in practice	0.000	1.00	0.990
What percentage of your daily work is reading diagnostic studies			
> *75%*	9.697	16,267.80	0.997
*25–49%*	−0.657	0.52	0.655
*50–75%*	4.337	76.46	0.745
*None*	−2.677	0.07	0.070
What percentage of your time is dedicated to administrative non clinical duties			
*25–49%*	1.565	4.78	0.346
*None*	−0.937	0.39	0.519

^*^Significant result. To resolve a false positive problem in the multiple testing, Bonferroni correction was performed and the significance level was adjusted to *p* < 0.0036

**Table 7:** Multivariate logistic regression: global specialty satisfaction

	**Coefficient**	**Odds-ratio**	***p*-value**
Clinical guidelines restrict my freedom to practice	0.308	1.36	0.327
Gatekeeping requirements seldom conflict with my clinical judgment	−0.411	0.66	0.427
I am able to refer patients or receive referrals when necessary	−0.043	0.96	0.896
Outside reviewers rarely question my professional judgements	−0.102	0.90	0.755
Personal time	0.129	1.14	0.762
Relationships with patients	2.185	8.89	0.001*
Relationships with colleagues	1.081	2.95	0.074
Relationships with staff	0.272	1.31	0.566
Pay	−0.061	0.94	0.867
My role in managing the business aspects of my practice is not a burden to me	0.543	1.72	0.079
Paperwork required by papers is a burden to me	0.095	1.10	0.754
I have too much administrative work to do	−0.300	0.74	0.347
Medical supplies are available when I need them	0.307	1.36	0.315
There are not enough support staff in my practice	0.226	1.25	0.461
Current level of practice (practicing interventional radiologist)	−0.649	0.53	0.713
Gender (Male)	0.391	1.48	0.637
Ethnicity (Others)	0.056	1.06	0.956
Ethnicity (White)	−0.398	0.67	0.671
Location of Practice			
*Northeast*	−1.331	0.26	0.191
*Southeast*	−0.235	0.79	0.809
*Southwest*	−1.103	0.33	0.439
*West*	0.361	1.43	0.731
Description of practice			
*Hybrid* + *Other*	0.925	2.52	0.403
*Private*	−0.381	0.68	0.670
Practicing in more than one hospital setting (Yes)	−0.984	0.37	0.198
Having an institutional practice leadership position (Yes)	−0.080	0.92	0.907
How many years have you been in practice	0.015	1.01	0.752
What percentage of your daily work is reading diagnostic studies			
> *75%*	13.847	1,031,846.7	0.997
*25–49%*	0.746	2.11	0.418
*50–75%*	−17.711	0.00	0.991
*None*	0.028	1.03	0.974
What percentage of your time is dedicated to administrative non clinical duties			
*25–49%*	1.361	3.90	0.270
*None*	0.182	1.20	0.854

^*^Significant result. To resolve a false positive problem in the multiple testing, Bonferroni correction was performed and the significance level was adjusted to *p* < 0.0036

### Qualitative analysis

Twenty-four participants (21.2%) provided comments in the optional free-text box at the conclusion of the survey; respondents noted administrative burden and “turf wars” with other specialties as factors negatively impacting their satisfaction.

## Discussion

This study demonstrated variable professional fulfillment among interventional radiologists. The mean job, career, and specialty satisfaction scores were 3.48, 3.40, and 3.62, respectively. Within these individual component measures, mean job and career satisfaction fell under the reported range of averages across a prior multispecialty study (3.52–3.81 for job satisfaction and 3.55–3.80 for career satisfaction) [18]. Contrastingly, specialty satisfaction amongst interventional radiologists was on par with the highest reported specialties in the prior study (3.17–3.76). Importantly, this study was from 20 years earlier, and significant interim changes in the practice of interventional radiology and clinical medicine may limit direct comparison. However, this divergence suggests that respondents are committed to their chosen field, but they also may experience challenges in the daily realities of practice that take away from their overall feeling of professional fulfillment.

More granular analysis with multivariate analysis revealed that relationships with patients were positively correlated with all three composite aspects of professional fulfillment. While consistent with research in other specialties [19], this is particularly salient within radiology as it may explain fundamental differences in professional fulfillment between diagnostic and interventional radiology. This is also a finding that might distinguish this subspecialty from diagnostic radiology and guide current and future efforts to enhance engagement and satisfaction within similar specialties. Personal time was correlated with both job and career satisfaction, and pay was correlated with career satisfaction. This is consistent not only with prior radiology-based studies [20, 21], but also non-radiologic specialty-specific research [22]. While perhaps unsurprising that work-life balance is a large factor, this bears reflection from practice and societal leadership moving forward. There is existing literature pertaining to work-life balance and wellness that found lower self-reported scores in those categories for IR physicians [23]. Strategies to improve wellness among the specialty might focus on professional role and identity, maintaining supportive workplaces, and providing resources for both physical and emotional well-being, including mentorship, stress management, and peer support.

Further, some qualitative responses to the survey noted administrative burdens and “turf wars” between specialties. This finding, while restricted to only a few survey respondents, suggests a systemic issue external to the individual interventional radiologist. There are several societal and interpersonal approaches to minimizing these types of conflicts, including the “able, affable, and available” mindset to better communicate interprofessionally. Societally, guidelines pertaining to accurate dictation for procedures, quality measurements for patient care, and patient awareness campaigns regarding the role of IR in patient care might be further employed to better address these concerns.

Direct comparison with previous studies of radiologist satisfaction is limited given methodological differences—prior studies’ results constitute a wide range of reported satisfaction scores within the three reported domains [20–22, 24]. However, prior studies have reported low wellness scores amongst practicing interventional radiologists, as well high degrees of moral injury which has been defined as a transgression of individual ethical values at a systemic or institutional level [13]. These two findings, when combined, may account for the discrepancy between the relatively low job and career satisfaction scores and the high specialty satisfaction scores. These findings deserve further investigation, as not only have low job satisfaction scores been correlated with physician burnout amongst radiologists, [25, 26], but overall levels among providers have been increasing in recent years and particularly accelerated by the recent Covid-19 pandemic [11, 12, 27]. These findings carry implications not only for the interventional radiology workforce, but secondarily health system care delivery and patient outcomes.

There are several limitations of this study. First, the survey population may be impacted by selection bias and may not be reflective of the general population of interventional radiologists. Prior work has demonstrated 27% of physicians in IR practice in an academic setting, while the sample here found that number to be 37.7%. Our sample also had 43.4% of IR physicians in a private setting, while that number has been found to be above 50% in the literature [28]. Related to this, the work herein was based in the United States, leaving out potential participants on other continents, such as Europe. Also, the small proportion of interventional radiology trainees in this study limits specific insights into this group. Furthermore, both the survey design and small sample population limited this survey’s ability to provide insight into job satisfaction among different groups. Indeed, some previous studies of job satisfaction among physicians have shown variations by race and gender [20, 29–33], though this has not been consistently demonstrated [21, 34]. Further research is needed to further validate recent trends in interventional radiologist satisfaction, as well as address the limitations above.

Overall, the current study demonstrates that while interventional radiologists may derive professional fulfillment from satisfaction with their specialty choice, their relatively low scores in job and career satisfaction have important implications for the field and healthcare delivery. Given the findings discussed here and the paucity of literature on this topic of professional fulfillment in a rapidly evolving and expanding subspecialty, further research is needed to better describe the specific environmental, institutional, and interpersonal factors that influence satisfaction across career stages, practice models, and demographics. More inclusive studies, building on the findings here, are warranted to explore how gender, race, and training status intersect with professional fulfillment in interventional radiology.

## Conclusions

This study provides one of the first targeted assessments of professional fulfillment among interventional radiologists using a validated, specialty-agnostic assessment tool. The findings demonstrate that while approximately half of respondents reported being satisfied with their specialty, minority proportions expressed satisfaction with their current job and career trajectory. Patient interactions and work-life balance were positive effectors of professional fulfillment. The quantitative and qualitative findings here not only guide future research but also highlight actionable areas that can be addressed in the near term, including interventions aimed at adjusting personal time and promoting more patient-centered roles, as well as more systemic change such as minimizing administrative hurdles and clarifying scopes of practice. Addressing the factors discussed herein and how they relate to professional fulfillment is not only critical to workforce well-being but may also yield downstream benefits for patient care and health system performance. 

## Data Availability

Not applicable.

## Appendix

### Survey Instrument

1. What describes your current level of practice?
2. What is your gender?
3. What is your race/ethnicity? You may select multiple options
4. Where do you practice?
5. What best describes your practice?
6. Do you practice in more than one hospital/setting?
7. Do you have an institutional/practice leadership position (e.g. section chief, department head)
8. (Trainees only) What is your current level of training?
9. (Practicing IRs only) How many years have you been in practice?
10. (Practicing IRs only) What percentage of your daily work is reading diagnostic studies (ie, non-procedural radiology)?
11. (Practicing IRs only) What percentage of your time is dedicated to administrative/non-clinical duties?
12. Clinical guidelines restrict my freedom to practice
13. Outside reviewers rarely question my professional judgements
14. I am able to refer patients or receive referrals when necessary.
15. Gatekeeping requirements seldom conflict with my clinical judgment.
16. Work rarely encroaches on my personal time.
17. My work schedule leaves me enough time for my family life.
18. The interruption of my personal life by work is a problem.
19. The amount of call I am required to take is not excessive.
20. I feel a strong personal connection with my patients.
21. The gratitude displayed by my patients keeps me going.
22. My relationship with patients is more adversarial than it used to be.
23. I am overwhelmed by the needs of my patients.
24. Many patients demand potentially unnecessary treatments.
25. Time pressures keep me from developing good patient relationships.
26. My physician colleagues are a good source of professional stimulation.
27. I get along well with my physician colleagues.
28. My physician colleagues value my unique perspective in practice.
29. My physician colleagues are an important source of personal support.
30. Nonphysicians in my practice support my professional judgment.
31. My nonphysician colleagues are a major source of personal support.
32. Nonphysician staff in my practice are not accommodating.
33. Nonphysicians in my practice reliably carry out clinical instructions.
34. My total compensation package is fair.
35. I am not well compensated given my training and experience.
36. I am not well compensated compared to physicians in other specialties
37. My role in managing the business aspects of my practice is not a burden to me.
38. Paperwork required by payers is a burden to me.
39. I have too much administrative work to do.
40. Medical supplies are available when I need them.
41. There are not enough support staff in my practice.
42. I find my present clinical work personally rewarding.
43. Overall, I am pleased with my work.
44. Overall, I am satisfied in my current practice.
45. My current work situation is a major source of frustration.
46. My work in this practice has not met my expectations.
47. If I were to choose over again, I would not become a physician.
48. All things considered, I am satisfied with my career as a physician.
49. In general, my medical career has met my expectations.
50. I would recommend medicine to others as a career.
51. Interventional radiology no longer has the appeal to me it used to have.
52. If I were to start my career over again, I would not choose interventional radiology.
53. I would recommend interventional radiology to a student seeking advice.
54. How did you hear about this survey?
55. (Optional) Any additional comments?

## Abbreviations

HIPAA: Health Insurance Portability and Accountability Act
IRB: Institutional Review Board
CFR: Code of Federal Regulations
STROBE: STrengthening the Reporting of OBservational studies in Epidemiology
